# SEFPN: Scale-Equalizing Feature Pyramid Network for Object Detection

**DOI:** 10.3390/s21217136

**Published:** 2021-10-27

**Authors:** Zhiqiang Zhang, Xin Qiu, Yongzhou Li

**Affiliations:** 1 Institute of Microelectronics, Chinese Academy of Sciences, No. 3 Beitucheng West Road, Chaoyang District, Beijing 100029, China; zhangzhiqiang2019@ime.ac.cn (Z.Z.); qiuxin@ime.ac.cn (X.Q.); 2 University of Chinese Academy of Sciences, No. 19(A) Yuquan Road, Shijingshan District, Beijing 100049, China

**Keywords:** object detection, feature pyramid, level imbalance, correlation convolution

## Abstract

Feature Pyramid Network (FPN) is used as the neck of current popular object detection networks. Research has shown that the structure of FPN has some defects. In addition to the loss of information caused by the reduction of the channel number, the features scale of different levels are also different, and the corresponding information at different abstract levels are also different, resulting in a semantic gap between each level. We call the semantic gap level imbalance. Correlation convolution is a way to alleviate the imbalance between adjacent layers; however, how to alleviate imbalance between all levels is another problem. In this article, we propose a new simple but effective network structure called Scale-Equalizing Feature Pyramid Network (SEFPN), which generates multiple features of different scales by iteratively fusing the features of each level. SEFPN improves the overall performance of the network by balancing the semantic representation of each layer of features. The experimental results on the MS-COCO2017 dataset show that the integration of SEFPN as a standalone module into the one-stage network can further improve the performance of the detector, by ∼1AP, and improve the detection performance of Faster R-CNN, a typical two-stage network, especially for large object detection $AP_{L}$∼2AP.

## 1. Introduction

**Figure 1:**
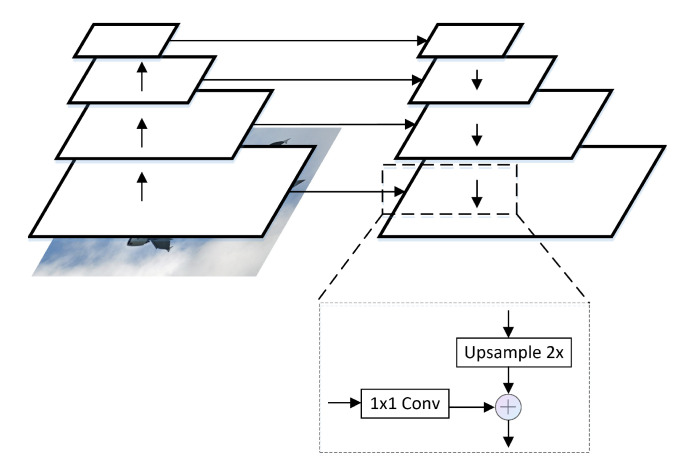
4-level FPN. For the reasons of share head and feature fuse, FPN’s output features have the same channel number but different scale.

It is a challenging task to detect objects of different scales in the field of computer vision. With the development of deep convolutional networks, many detectors have made significant improvements in object detection [1,2]. In order to extract effective features, the detector can use powerful network structures such as ResNet [3], HourglassNet [4], HTC [5], etc. Using them as backbone, two-stage detectors [6,7] and one-stage detectors [8,9,10] lead to improvements in detection performance and speed, respectively. On this basis, Anchor Base [11,12,13] and Anchor-Free [14,15] detectors further improve the detection performance of the detectors. No matter what kind of strategy the detector adopts, the detection network is usually designed to consist of three parts: backbone, neck, and head. Feature Pyramid Network (FPN) [16] is a commonly used neck structure, as shown in Figure 1. The input feature features of FPN have the following characteristics: low-level features have high resolution and less channels, while high-level features have low resolution and more channels, but high-level features and low-level features are complementary [17]. The output features of the feature pyramid FPN have multiple scales but the same number of channels, and the features are fused through a top-down path. The head part uses the features output of FPN as input for object detection according to different strategies, such as FSAF [18] and ATSS [19].

As the features extracted from backbone are in different network depths, there is a semantic gap between the features. FPN adopts a top-down path to improve the semantic representation of the underlying features by sequentially fusing features from the upper-level, thereby improving the detection performance of the network.

From the output point of view, after the input feature passes through the FPN, the number of channels of the high-level feature is reduced from 2048 to 256, and then information loss appears [17]. At the same time, FPN merges the high-level features with the next-level features sequentially through a top-down path, so that the generated features pay more attention to adjacent resolution features and less attention to other features. In this way, high-level features are passed down. The semantic information contained in non-adjacent layers will be diluted after each cross-layer fusion. It does not make full use of the correlation [20] and complementarity between high-level and low-level features.

**Figure 2:**
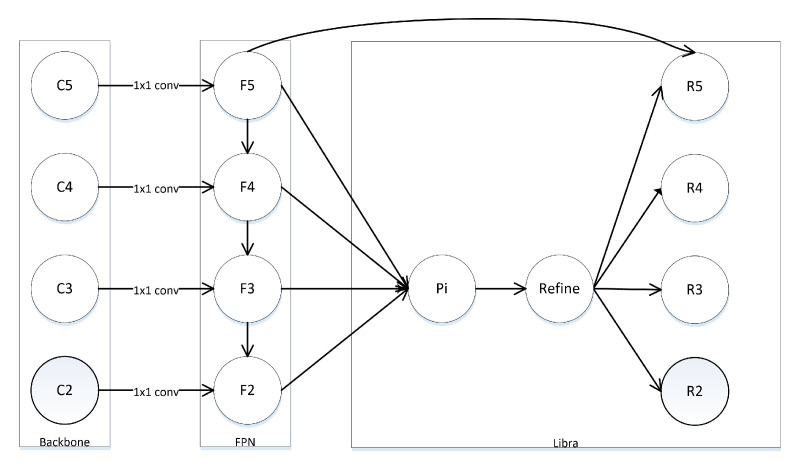
Libra RCNN’s BFP. BFP re-scale the output features of FPN into one size and use summation to fuse the features, then resize the output feature into different scale.

**Figure 3:**
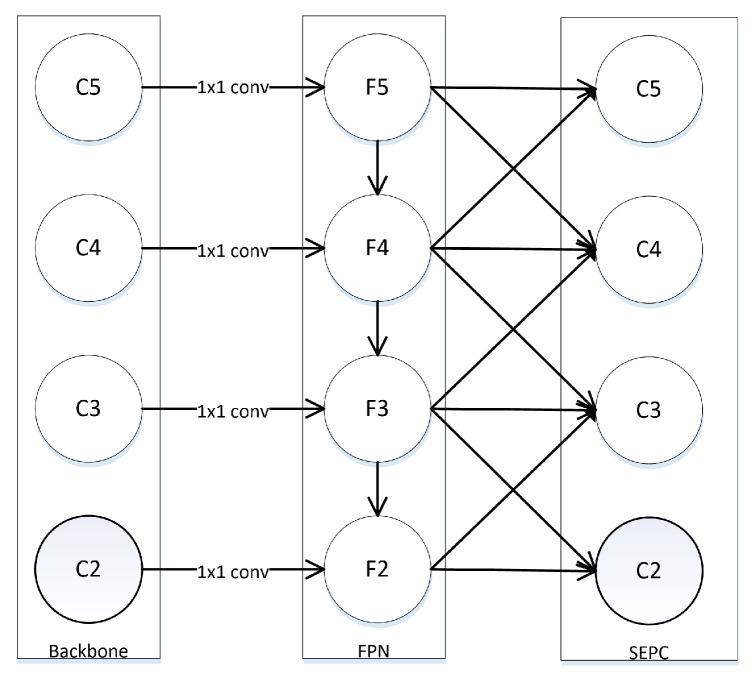
3D Convolution-SEPC. Pconv apply the correlation between adjacent feature layers to fuse the features. SEPC construct a layer which use PConv on all level of input features.

To solve the problem of semantic gap, new solutions were proposed. PANet [21] introduced a bottom-up path to pass the low-level features to the high-level. Libra R-CNN [22] proposed the Balanced Feature Pyramids(BFP) which combines the FPN output features of different levels into a middle scale, and then generates features of different sizes through rescaling, as shown in Figure 2. The way of processing features in BFP is similar to image pyramid and SNIP [23,24]. X. Wang proposed to use a 3D convolution, called Scale-Equalizing Pyramid Convolution (SEPC) [20], to extract scale and spatial features simultaneously, as shown in Figure 3. SEPC mainly focuses on the inter-level correlation in the feature pyramid but ignores the complementarity of high and low layer features.

Inspired by SEPC and Libra R-CNN, we propose a simple but effective method, namely, Scale-Equalized Feature Pyramid Network (SEFPN), to deal with semantic gaps. Through the iterative fusion of features with various resolutions, the semantic difference of output features is reduced. To evaluate the proposed method, we validated it on the MS-COCO2017 dataset. Using the FoveaBox method [14], a one-stage method, SEFPN can improve the Average Precision (AP) by 1%, especially for large scale object detection ($AP_{L}$) by 2%. Experiments on Faster R-CNN, a two-stage algorithm, show a 2% improvement in large object detection ($AP_{L}$). These experiments fully prove that SEFPN is an effective way to improve the efficiency of network detection.

## 2. Related Work

### 2.1. Deep Object Detector

Modern deep detection networks use convolutional (CNN) neural networks to detect objects. Object detection methods can be divided into two-stage and one-stage according to whether to they use the Region Proposal Network (RPN) [25]. Two-stage detectors, such as Faster R-CNN [7] and its improvements, use RPN to extract the Region Of Interest (ROI), and then classify and locate the ROI. One-stage detectors, e.g., YOLO [9], follow an end-to-end manner to classify and locate objects on features. Compared with two-stage detectors, one-stage detectors are more efficient. RefineNet [26] introduces task-specific branches into the network architecture to surpass the accuracy of the two-stage detector while it maintains the speed of the one-stage detector.

According to whether an anchor is used or not, it can be divided into anchor-free and anchor base algorithms. The anchor base algorithm is used to layout priori anchor boxes on the features. The anchor box parameters depend on the dataset used. Typical algorithms are YOLOV3 [10], SSD [13], etc. While the anchor-free algorithm avoids the use of anchor, the entire framework has a lower computational cost and runs faster, such as FoveaBox [14] and CornerNet [15]. ATSS [19] adopts a new positive sample sampling strategy to bridge the gap between anchor-based and anchor-free detection.

This paper uses FoveaBox as the baseline, which is an anchor-free, one-stage detector. It detects objects on features of different scales according to the size of the object, such as SNIP [23]. If the center of the sample is in a specific smaller area, this sample is selected as positive.

### 2.2. Feature Fusion

The output of FPN is a feature set with multiple levels, but the same number of channels, and can apply different object detection strategies. The FPN structure has the following defects: on the one hand, high-level features suffer from information loss due to the reduced feature channels. On the other hand, a top-down path transfers high-level features to the bottom through sequential fusion. The sequential approach will allow the integrated feature to focus more on adjacent resolutions rather than other resolutions. During the information flow, the semantic information contained in non-adjacent layers will be diluted once every time when they are fused. The result is that there is a semantic gap between high-level features and low-level features, and FPN does not make full use of the complementarity between features.

PANet [21] adds a bottom-up path on FPN in an attempt to improve the content descriptive of high-level features. Libra R-CNN [22] integrates the output of FPN into a specific scale feature, and then re-scales it into different scale features through upsampling and downsampling. Its main idea is to lighten up the semantics gap.

SEPC [20] applies the correlation between adjacent-layer features to fuse those features using a 3D convolution method. AugFPN [17] introduces Residual Feature Augmentation (RFA) and Adaptive Spatial Fusion (ASF) to improve network performance and reduces the influence of FPN network defects. NasFPN [27] uses Neural Architecture Search(NAS) to search for a better network structure, which greatly improves the detection results. ASFF [28] generates weight coefficients for the fusing features and adopts the adaptive strategy for feature fusion.

Inspired by Libra R-CNN and SEPC, this paper proposes a new network structure called Scale-Equalizing Feature Pyramid Network(SEFPN). SEFPN can generate balanced features while considering the correlation between features of different scales, and the computational cost will maintain in a low increase.

## 3. Framework

### 3.1. Overall

**Figure 4:**
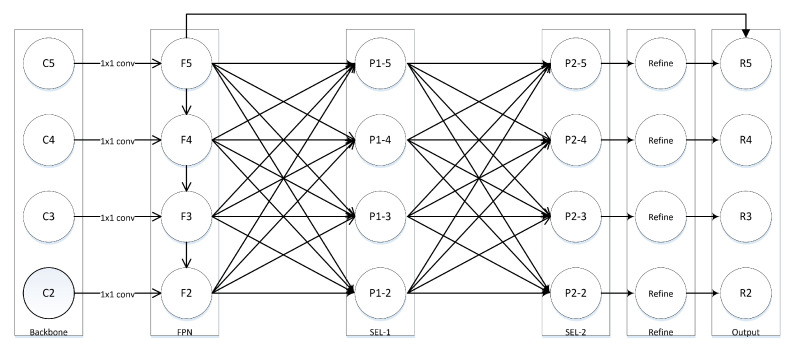
Pipeline of SEFPN. The whole pipeline is constructed by FPN, full connected layer, and a refine module. FPN acts as a basic module to output features. The full connected layer acts as a module to fuse all scales features. The last module is used to make the feature coarse or fine.

The whole framework pipeline is as follows. First, features with channel number set to [256, 512, 1024, 2048] are extracted from backbone. In other words, the scale of high-level feature is 1/2 of the adjacent low-level feature scale, that is, $Scale_{C_{i}}=\frac{1}{2}Scale_{C_{i-1}}$. Second, FPN takes the output feature as input, then feature $F_{i}$ with a channel number of 256 as output. Feature $F_{i}$ is rescaled to generate a specific size and then fused with other features to form one level $P_{i,j}$, then put all levels together to form one stage. Finally, the output feature will pass a refine module. The pipeline of framework is shown in Figure 4.

### 3.2. Multi-Level Libra

To balance the feature representation, Libra R-CNN proposes to re-scale the output features of FPN into a specific scale. The refine method is applied through the non-local module, and the final re-scale will generate features $R_{i}$ of different scales. That is, the only difference between the features $R_{i}$ is the scale.

The proposed SEFPN is an improvement of Libra RCNN. Because of the complementary relationship between high-level features and low-level features, as well as the correlation between feature layers, directly integrating features at all levels is undoubtedly the most straightforward way. Thus, features in $Level_{i}$ can be expressed as

(1) $$y^{i}=\sum_{j=i+1}^{j=L}Upsample\left(x^{j}\right)+x^{i}+\sum_{j=0}^{j=i-1}Downsample\left(x^{j}\right)\;$$

In SEFPN, the upsampling method uses bilinear interpolation, and the downsampling method uses global max pooling. Compared with the SEPC, it just merges feature from $Level_{i}$ with those from adjacent levels $Level_{i-1}$, $Level_{i+1}$, that is,

(2) $$y^{i}=Upsample\left(\omega^{i+1}*x^{j}\right)+\omega^{i}*x^{i}+Downsample\left(\omega^{i-1}*x^{j}\right)\;$$

SEPC requires more parameters and more computational cost. In intuition, the output features still suffer from semantic gap between different levels.

### 3.3. Multi-Block Libra

Inspired by SEPC, we treat multi-level Libra as a block. In order to fully fuse the features of different scales, we arrange the blocks in a sequential manner to form a multi-block network. To prevent the gradient vanishing, a short connect is added between block input and the output. The block is formulated as (3).

(3) $$Output=x+f\left(y_{l}\right),y_{l}=y^{i}|i\in \left[0,L-1\right].\;$$

f(·) is the refine method.

### 3.4. Refine Method

The purpose of the refine method is to introduce an attention mechanism to make the generated features focus on a certain feature component according to the different inputs. We select Non-Local [29], GC Block [30], and convolution as candidates, and the experimental results show that SEFPN is not sensitive to the type of refine method.

## 4. Experiments

### 4.1. Dataset and Evaluation Metrics

All experiments in this paper are performed on the challenging MS-COCO2017 dataset [31]. The dataset contains 80 categories of around 160K images (118k images for training, 5k images for validation, and 41k images for testing). All reported results follow the standard COCO-style mean Average Precision (mAP) metrics under different IoU thresholds, ranging from 0.5 to 0.95. We also report the results $AP_{S}$, $AP_{M}$, $AP_{L}$ on small, medium, and large scales, respectively.

### 4.2. Implementation Details

All experiments are implemented based on mm detection [32]. The input images are first resized to (1333, 800). We perform our training using one compute node of 2 A100 GPUs each with 40GB memory. The initial learning rate is set as 0.0125. In the training process, 1× schedule means 12 epochs, and 2× schedule means 24 epochs. In 1× scheduling, the learning rate drops by 0.1 after 8 and 11 epochs, respectively, and 2× scheduling drops after 16 and 22 epochs. All other parameters not noted in this paper following mm detection. We chose the one-stage detector FoveaBox and two-stage detector Faster RCNN as baseline.

### 4.3. Main Results

To verify the effectiveness of the SEFPN method for performance improvement, we evaluate SEFPN on COCO test set and compare it with other SOTA one-stage and two-stage detectors. For a fair comparison, we have re-implemented the corresponding baseline methods with FPN on mm detection. All results are shown in Table 1. By appending SEFPN after FPN, when using ResNet50 and ResNet101 as backbone, Faster R-CNN can achieve 37.3AP and 39.6AP, respectively, which is higher than that on ResNet50 with FPN. Specifically, Faster R-CNN with SEFPN can achieve 47.7AP and 52.0AP on large-scale object detection, which is 3.1AP and 3.7AP higher than Faster R-CNN with FPN. Obviously, Faster R-CNN with SEFPN is better than Faster R-CNN with FPN on large-scale object detection.

For one-stage detectors, we append SEFPN after FPN in FoveaBox detector. FoveaBox with SEFPN boosts all the metrics by almost 1AP. When using ResNet50 and ResNet101 as backbone, FoveaBox with SEFPN brings AP to 37.3 and 39.1. when 2× schedule is adopted, FoveaBox with SEFPN and ResNet101 can achieve 52.6AP on large-scale object detect, which is 2AP higher than FoveaBox with FPN.

As shown in Table 1, detector with SEFPN can boost the detect performance, especially for the detection of large-scale objects. Because SEFPN performs better on large-scale object detection, we attribute this to the information loss of high-level features, while high-level features are used for small-scale object detection in FoveaBox with SEFPN. For Faster R-CNN with SEFPN, in addition to the information loss, the refinement module might be also considered.

### 4.4. Computing Costs

Table 2 compares the changes of FPS, FLOP, and parameters after the Faster RCNN algorithm and the Foveabox algorithm modify the NECK structure. After modifying Faster RCNN, FPS dropped from 5.3 to 4.8, ~9%. The computational complexity(FLOP and Parameters) is very small and can be ignored. After Foveabox replaced FPN with SEFPN, FPS dropped by 0.8. The FLOP has increased by 5.6 G, which is about 2.7%. The rate of increase in the calculation cost is acceptable.

### 4.5. Ablation Study

#### 4.5.1. Effectiveness of the Block Number

To evaluate the impact of different block numbers on the performance of detector, we set the framework as following, using ResNet50 as Backbone, FPN as Neck, and set the number of blocks to be 1, 2, and 4, respectively, and use the refinement method Non-Local. Each block shares the same refinement method, and the number of nodes in each block is set to be 5.

Table 3 shows the experimental results. It can be seen from the table that setting the block number as 2 has the best effect. From the point of view of network structure, each block is a direct fusion of input features. After two fusions, the output features can be considered to have reached an equilibrium state.

#### 4.5.2. Effectiveness of Batch Normalization Method

During experiments, we found that adding an integrated Batch Normalization (BN) method can improve the performance. BN_pre places BN method before the refine method, while BN_post places BN after the refine method. Other settings are as follows. Backbone is ResNet50, fusion block number is 2, and refine method is Non-Local.

Table 4 shows the experimental results. Refine method acts as a self-attention module. While in BN_pre setting, BN normalizes the input feature of refine module, which indirectly weakens the function of refine. BN_post gets a better result, brings all metrics up.

#### 4.5.3. Effectiveness of Refine Method

To evaluate the impact of different refine method, we design experiments in this section on Non-Local, global context (GC Block) [30], and 3 × 3 convolution block. We set the backbone as ResNet50, FPN as Neck, and set the number of block as 2. Compared with no refinement setting, using Non-Local and GC Block can improve the detection metrics.

The results have shown in Table 5. Especially, Non-Local gets 1AP up on large-scale object detection, GC Block has achieved an improvement of 1.6AP on small scale object. Surprisingly, convolution can also achieve similar results. GC Block+ is not good as GC Block because of putting more attention on all level features.

## 5. Discussion

We define feature imbalance as the difference in object detection ability between feature layers. That is, deep, high-level features in backbones have more semantic meanings, while the shallow low-level features are more content descriptive. The imbalance may just because the features are at different levels, or the distribution of object categories and sizes of the data set, or the ratio of positive and negative samples at each level. This article discusses imbalance between feature levels, which is due to the fact that the deeper the backbone network, the more semantics; on the contrary, the less location information. FPN introduce a top-down path to make the semantic information in high-level flows to low-level features. Moreover, PANet adds a bottom-up path to introduce the position information of the low-level into the high-level features.

Through FPN and PANet, the object detection performance of the network is improved. As shown in Figure 1, FPN uses a sequential method to fuse high-level and low-level feature, which will cause information attenuation. That is, feature in lower level get less semanticinformation. Therefore, what is the best way to make the lower-level feature more semantic? The answer is to directly fuse the high-level and low-level features, as described in this paper. By comparing with Balance FPN, both algorithms use the direct fusion of all level features. The difference is that Balance FPN fuses features into a single size, and then features of different sizes are generated by resize. Our method directly merges features of different sizes, and at the same time, ensures that the output features are fully fused through iterative operations. The experimental results in Table 3 show that multiple layers fusions is better than single layer fusions. We believe that the features have been fully integrated, and the imbalance between features has been eliminated to a certain extent.

For the postprocessing of the output features, as Table 4 and Table 5 show, we verified the impact of using BN and Self-attention on the overall performance. Integrate BN works on a 3D convolution in PConv, which is similar to what we do in this paper. As the results shown in SEPC, iBN can also play a role on multi-layer features fusion.

As for the Non-Local and GC Block of Self-Attention, Table 5 shows that the overall performance has been improved. Due to the computational cost of Self-Attention, we evaluated the increase in computational cost, with results shown in Table 2. As can be seen from network structure, the previous multi-layer libra part uses linear interpolation and does not require parameters. The network parameters are mainly concentrated in the refinement part. In order to reduce parameters, we share the method between levels. That is, each level uses the same attention module. These make the overall calculation load a little increase.

We have verified through experiments that the direct fusion of features in our way can improve the detection performance of the network, but in fact, there is still no way to quantitatively measure the specific difference in semantics and positioning information between the feature layers. Although it is possible to use Loss and IoU, as well as positive and negative sample ratios, etc., these require samples to go through a complete pipeline. We are exploring a new way to directly measure the imbalance of features. This is also our later work.

## 6. Conclusions

In this paper, we analyze the imbalance problem of different scale features along with FPN, and present a novel object detection neck, which generates multiple features of different scales by iteratively fusing features of each level. With the help of simple but effective components, we alleviate the semantic gap of different level features generated by FPN. Through the improvement on small-scale objects is not so obvious, with less computation cost, it still improves 1mAP on Foveabox. On the other hand, it bring up a 2mAP on Faster-RCNN. It can be used as a plugin block and integrated into existing detector frameworks to improve their performance. In the future work, we will verify the generalization of SEFPN on more detectors.

## Figures and Tables

**Table 1:** Comparison with the state-of-the-art results on MS-COCO, symbol “*” means our re-implemented results.

Method	Backbone	Epoch	AP	${\mathrm{AP}}_{50}$	${\mathrm{AP}}_{75}$	${\mathrm{AP}}_{S}$	${\mathrm{AP}}_{M}$	${\mathrm{AP}}_{L}$
One-stage								
FoveaBox * [14]	ResNet50	1×	36.2	56.1	38.5	20.4	39.7	46.2
FoveaBox * [14]	ResNet101	1×	38.3	58.3	40.9	21.4	42.4	50.0
FoveaBox * [14]	ResNet101	2×	38.9	58.7	42.0	22.1	42.8	50.6
YOLOV2 [33]	Darknet19	-	21.6	44.0	19.2	5.0	22.4	35.5
YOLOV3 [10]	Darknet53	-	33.0	57.9	34.4	18.3	35.4	41.9
RetinaNet [34]	ResNet101	-	39.1	59.1	42.3	21.8	42.7	50.2
FCOS * [35]	ResNet50	1×	37.0	56.6	39.4	20.8	39.8	46.4
SSD512 [13]	ResNet101	-	31.2	50.4	33.3	10.2	34.5	49.8
CARAFE [36]	ResNet50	-	38.1	60.7	41.0	22.8	41.2	46.9
Two-stage								
Faster RCNN * [7]	ResNet50	1×	36.5	58.7	39.1	21.5	39.7	44.6
Faster RCNN * [7]	ResNet101	1×	38.9	60.9	42.3	22.4	42.4	48.3
Faster RCNN * [7]	ResNet101	2×	39.7	61.4	43.3	22.3	42.9	50.4
ours								
FoveaBox w/SEFPN	ResNet50	1×	37.3	58.0	39.6	22.0	41.0	47.7
FoveaBox w/SEFPN	ResNet101	1×	39.1	59.5	41.7	22.9	43.0	50.6
FoveaBox w/SEFPN	ResNet101	2×	39.9	61.0	42.8	23.3	43.8	52.6
Faster RCNN w/SEFPN	ResNet50	1×	37.3	58.6	40.6	22.2	40.3	47.7
Faster RCNN w/SEFPN	ResNet101	1×	39.6	60.6	43.4	22.8	43.5	52.0
Faster RCNN w/SEFPN	ResNet101	2×	39.7	60.2	43.6	22.3	43.2	52.5

**Table 2:** Compare the computing cost of Fast RCNN and Foveabox.

Method	FPS	FLOP(G)	Params(M)
Faster RCNN/w FPN	5.3	207.07	41.53
Faster RCNN/w SEFPN	4.8	207.17	41.57
Foveabox/w FPN	7.3	206.3	36.19
Foveabox/w SEFPN	6.5	211.9	36.46

**Table 3:** Ablation studies of block number on COCO val2017.

Block	AP	${\mathrm{AP}}_{50}$	${\mathrm{AP}}_{75}$	${\mathrm{AP}}_{S}$	${\mathrm{AP}}_{M}$	${\mathrm{AP}}_{L}$
1	37.0	57.5	39.2	22.1	40.9	47.1
2	37.3	57.9	39.3	21.8	41.1	48.5
4	36.9	57.8	38.9	21.7	41.0	47.2

**Table 4:** Ablation studies of BN method on COCO val2017.

Type	AP	${\mathrm{AP}}_{50}$	${\mathrm{AP}}_{75}$	${\mathrm{AP}}_{S}$	${\mathrm{AP}}_{M}$	${\mathrm{AP}}_{L}$
None	37.0	57.5	39.2	21.2	40.9	47.1
BN_pre	36.1	57.2	38.0	21.0	40.3	46.3
BN_post	37.3	58.0	39.6	22.0	41.0	47.7

**Table 5:** Ablation studies of refine method on COCO val2017.

Type	AP	${\mathrm{AP}}_{50}$	${\mathrm{AP}}_{75}$	${\mathrm{AP}}_{S}$	${\mathrm{AP}}_{M}$	${\mathrm{AP}}_{L}$
None	36.9	57.2	39.2	21.0	40.7	47.5
NonLocal	37.3	57.9	39.3	21.8	41.1	48.5
GC Block	37.2	57.9	39.2	22.6	41.1	47.2
GC Block+	36.9	57.4	39.2	21.9	40.9	47.4
Conv	37.1	57.1	39.5	20.5	40.7	48.4

## Data Availability

Not applicable.
